# Supplementary material for: Multiomics reveals persistence of obesity-associated immune cell phenotypes in adipose tissue during weight loss and weight regain in mice
Source: Nat Commun. 2022 May 26;13:2950. doi: 10.1038/s41467-022-30646-4 (PMC9135744; doi:10.1038/s41467-022-30646-4)
Supplement: Supplementary file 1 — Supplementary Information [file 41467_2022_30646_MOESM1_ESM.pdf]

SUPPLEMENTARY INFORMATION

Multiomics reveals persistence of obesity-associated immune cell phenotypes in adipose tissue during weight loss and weight regain in mice

Cottam, M.A., Caslin, H.L., Winn, N.C., and Hasty A.H.

TABLE OF CONTENTS

Supplementary Fig. 1: Interim body mass and ipGTT for male mice 1

Supplementary Fig. 2: Body mass and glucose tolerance for mice used for CITEseq analysis.. 2

Supplementary Fig. 3: Weight cycling in female C57BL/6J mice... 3

Supplementary Fig. 4: Validation of CITE-seq antibodies 4

Supplementary Fig. 5: Biological replicates identified by Hashtag demultiplexing are well representing within diet groups 5

Supplementary Fig. 6: Heatmap of top five differentially expressed genes for each high-resolution cluster identity 6

Supplementary Fig. 7: Frequency and markers of adipose tissue γ/δ, NK, and NKT cells.......... 8

Supplementary Fig. 8: Adipose tissue mast cells have reduced expression of *Ilr1l1* and ILC2s are reduced in frequency after obesity.. 9

Supplementary Fig. 9: Alignment of CD8+ T cells to a tumor-infiltrating lymphocyte reference atlas 10

Supplementary Fig. 10: Frequency and markers of adipose tissue B cells and plasma cells 11

Supplementary Table 1: Ordering information for antibodies and reagents..............................12

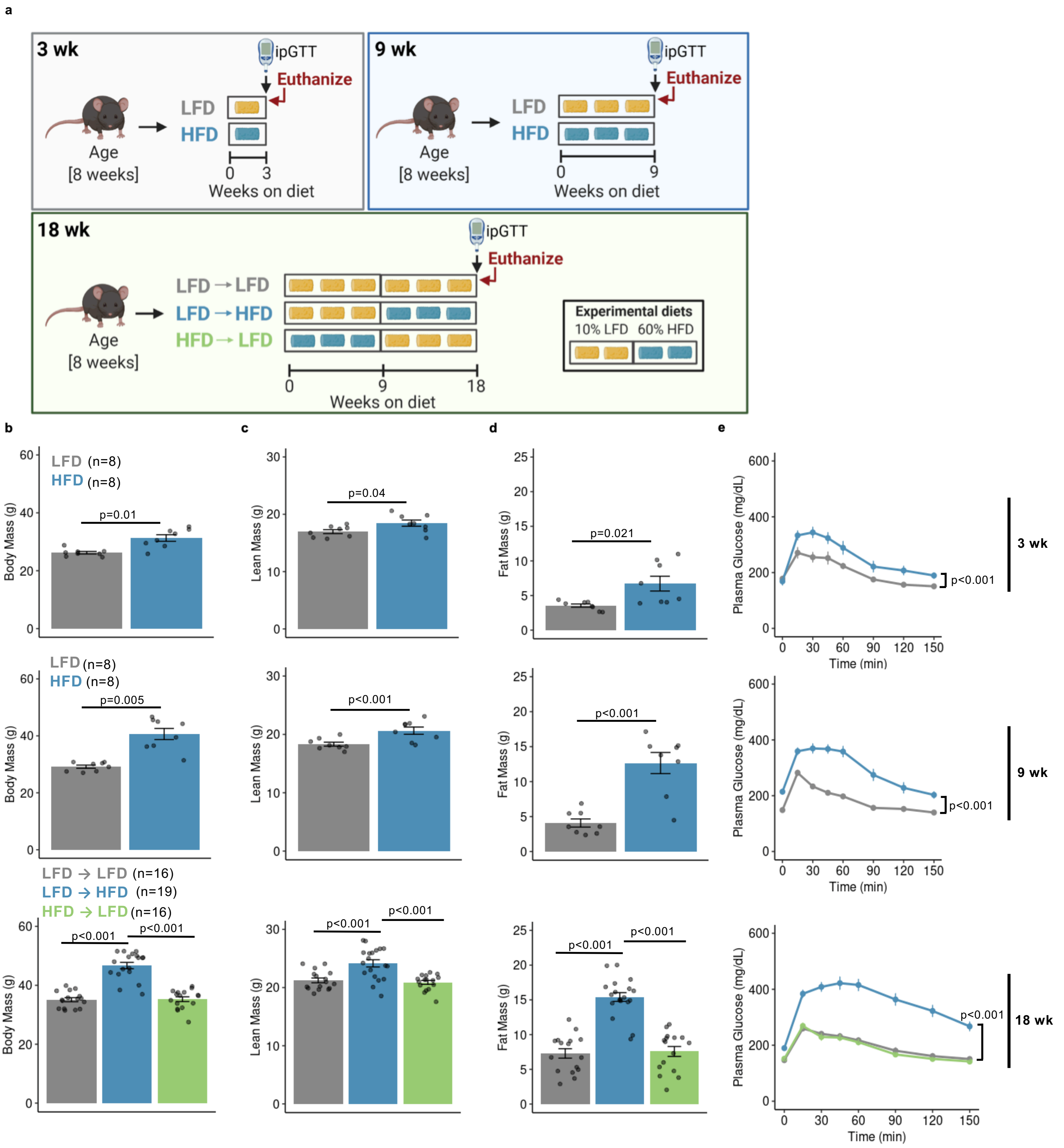

Supplementary Figure 1. Interim body mass and ipGTT for male mice. (**a**) Schematic of interim study timepoints. (**b**) Body mass, (**c**) lean mass, (**d**) fat mass, and (**e**) ipGTT at weeks 3, 9, and 18 for male mice (mean ± SEM). Pairwise two-tailed T-tests (3 and 9-week timepoints) with Bonferroni correction for multiple comparisons (18-week timepoint only) were used to compare groups for body mass, body composition, and ipGTT area under the curve. Significant p values are shown. Data are not paired, mice at each timepoint represent independent cohorts that were sacrificed at the indicated time.

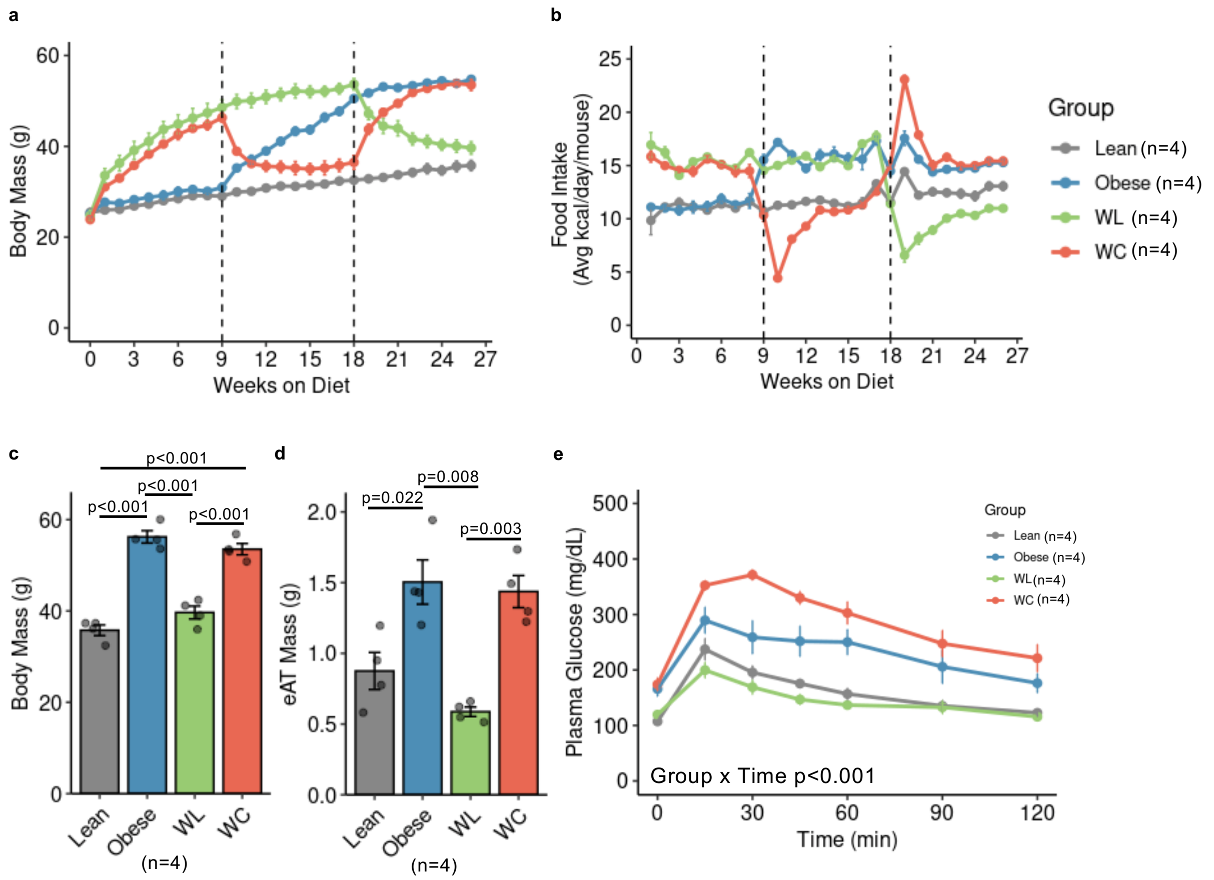

Supplementary Figure 2. Body mass and glucose tolerance for mice used for CITEseq analysis. (**a**) Body mass over time measured weekly. (**b**) Food intake over time measured weekly. (**c**) Body mass and (**d**) eAT mass measured at sacrifice. (**e**) Intraperitoneal glucose tolerance test dosed at 1.5 g dextrose/kg lean mass one week prior to sacrifice. Pairwise two-tailed T-tests with Bonferroni correction for multiple comparisons were used to compare groups for body mass and eAT mass and two-way ANOVA was used to compare groups for ipGTT; significant p values shown. Data are plotted as mean ± SEM (n=4 mice).

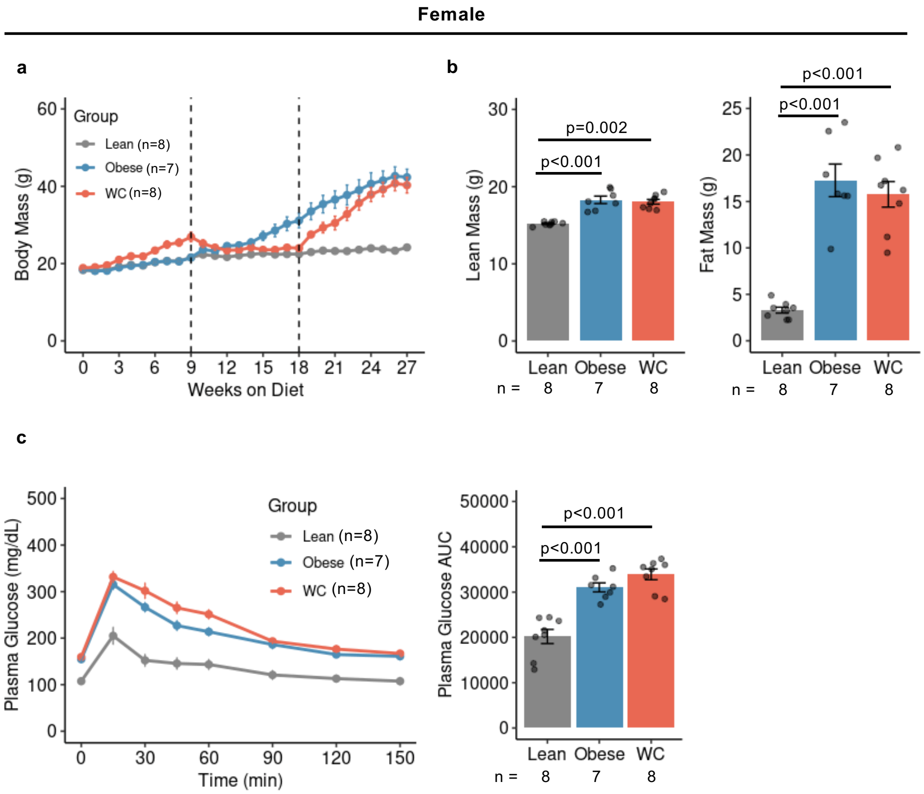

Supplementary Figure 3. Weight cycling in female C57BL/6J mice. (**a**) Body mass over time, (**b**) body composition after 27-weeks of diet, and (**c**) intraperitoneal glucose tolerance test and corresponding area under the curve (AUC) after 27-weeks of diet for female mice following our WC model (number of mice indicated for each panel; n). Pairwise two-tailed T-tests with Bonferroni correction for multiple comparisons were used to compare groups for body composition and ipGTT area under the curve; significant p values shown.

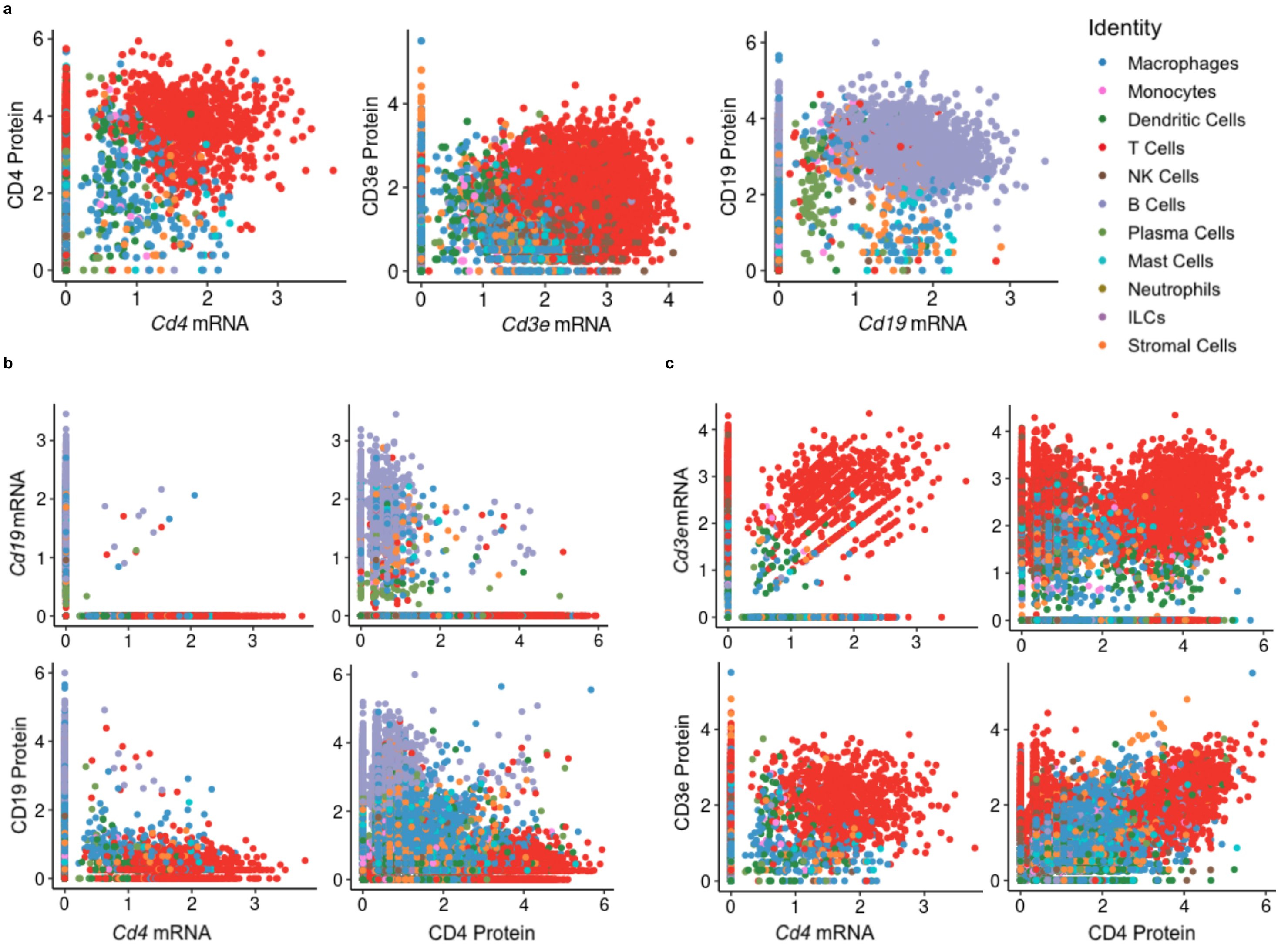

Supplementary Figure 4. Validation of CITE-seq antibodies. (**a**) Comparison of genes for *Cd3e, and Cd19* with their corresponding surface proteins measured using CITE-seq. Individual cells are colored by annotated cell type. (**b**) Comparison of cell type exclusive genes *Cd4* and *Cd19* with their corresponding surface proteins. (**c**) Comparison of co-expressed genes *Cd4* and *Cd3e* with their corresponding surface proteins.

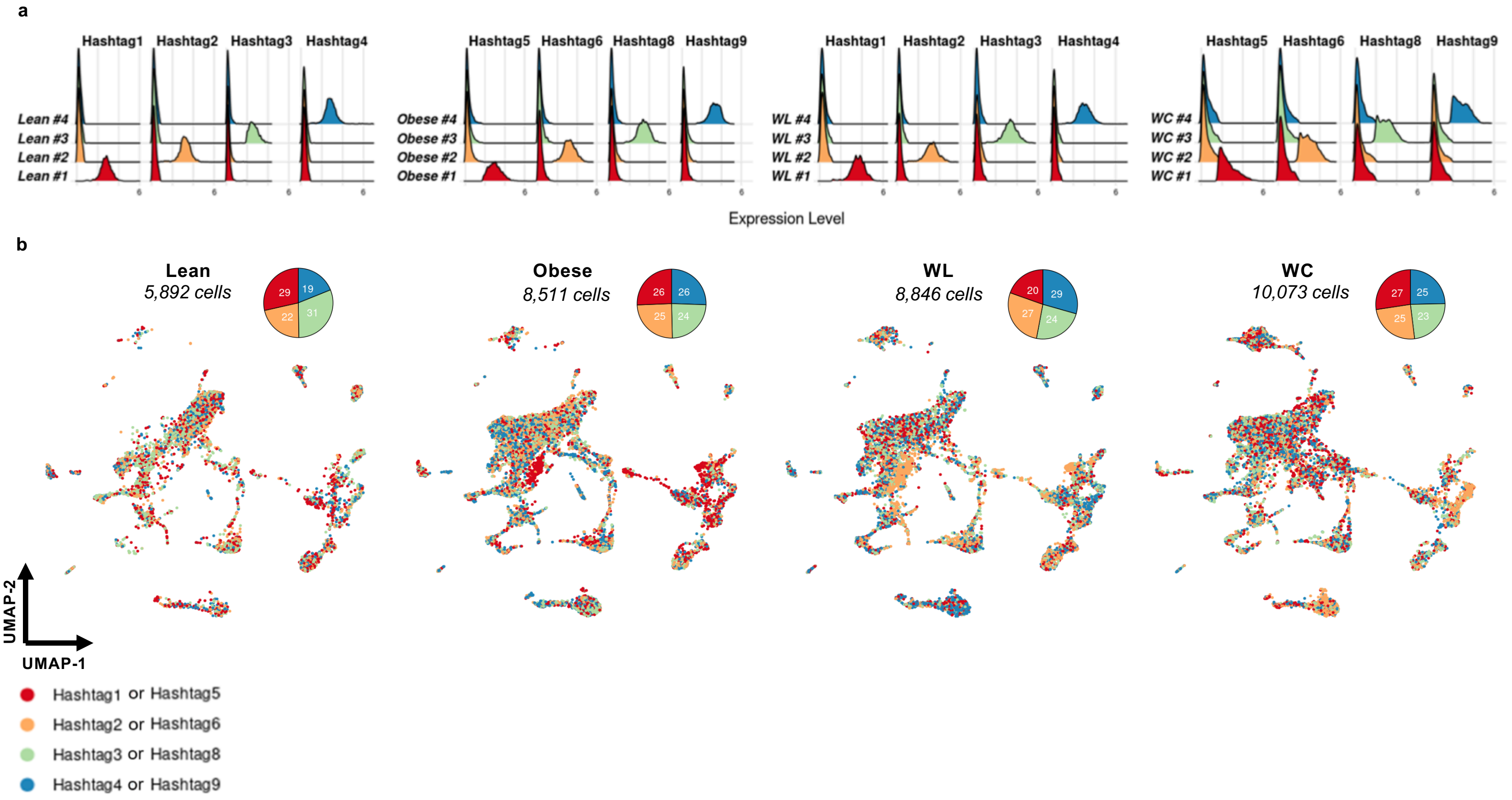

Supplementary Figure 5. Biological replicates identified by Hashtag demultiplexing are well represented within diet groups. (**a**) Hashtags demultiplexed for each individual mouse per diet group (n=4 per group; total of 16 mice sequenced). (**b**) Uniform manifold projection with total cell counts retained following QC for each diet group and distribution of hashtags measured within each diet group (inset).

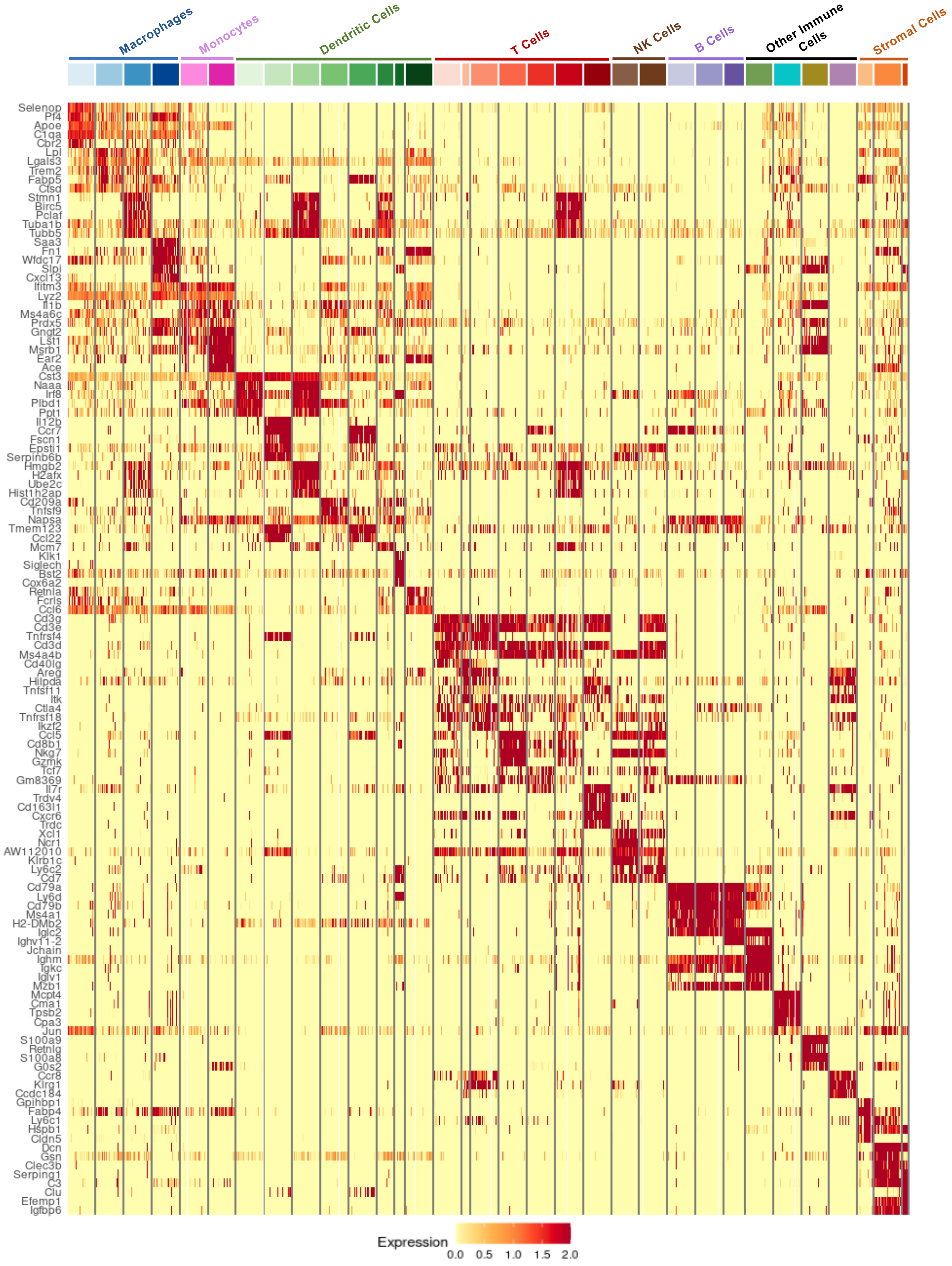

Macrophages
Monocytes
Dendritic Cells
T Cells
NK Cells
B Cells
Other Immune Cells
Stromal Cells
Selenop
Pf4
Apoe
C1qa
Cbr2
Lpl
Lgals3
Trem2
Fabp5
Ctsd
Stmn1
Birc5
Pclaf
Tuba1b
Tubb5
Saa3
Fn1
Wfdc17
Slpi
Cxcl13
Ifitm3
Lyz2
Il1b
Ms4a6c
Prdx5
Gngt2
Lst1
Msrb1
Ear2
Ace
Cst3
Naaa
Irf8
Plbd1
Ppt1
Il12b
Ccr7
Fscn1
Epsti1
Serpinb6b
Hmgb2
H2afx
Ube2c
Hist1h2ap
Cd209a
Tnfsf9
Napsa
Tmem123
Ccl22
Mcm7
Klk1
Siglech
Bst2
Cox6a2
Retnla
Fcrls
Ccl6
Cd3g
Cd3e
Tnfrsf4
Cd3d
Ms4a4b
Cd40lg
Areg
Hilpda
Tnfsf11
Itk
Ctla4
Tnfrsf18
Ikzf2
Ccl5
Cd8b1
Nkg7
Gzmk
Tcf7
Gm8369
Il7r
Trdv4
Cd163l1
Cxcr6
Trdc
Xcl1
Ncr1
AW112010
Klrb1c
Ly6c2
Cd7
Cd79a
Ly6d
Cd79b
Ms4a1
H2-DMb2
Iglc2
Ighv11-2
Jchain
Ighm
Igkc
Iglv1
Mzb1
Mcpt4
Cma1
Tpsb2
Cpa3
Jun
S100a9
Retnlg
S100a8
G0s2
Ccr8
Klrg1
Ccdc184
Gpihbp1
Fabp4
Ly6c1
Hspb1
Cldn5
Dcn
Gsn
Clec3b
Serping1
C3
Clu
Efemp1
Igfbp6
Expression
0.0 0.5 1.0 1.5 2.0

Supplemental Figure 6. Heatmap of top five differentially expressed genes for each high-resolution cluster identity. Each row is a unique gene, colored red when expressed highly. Each thin vertical line indicates one subsampled cell (up to 100 cells per subcluster).

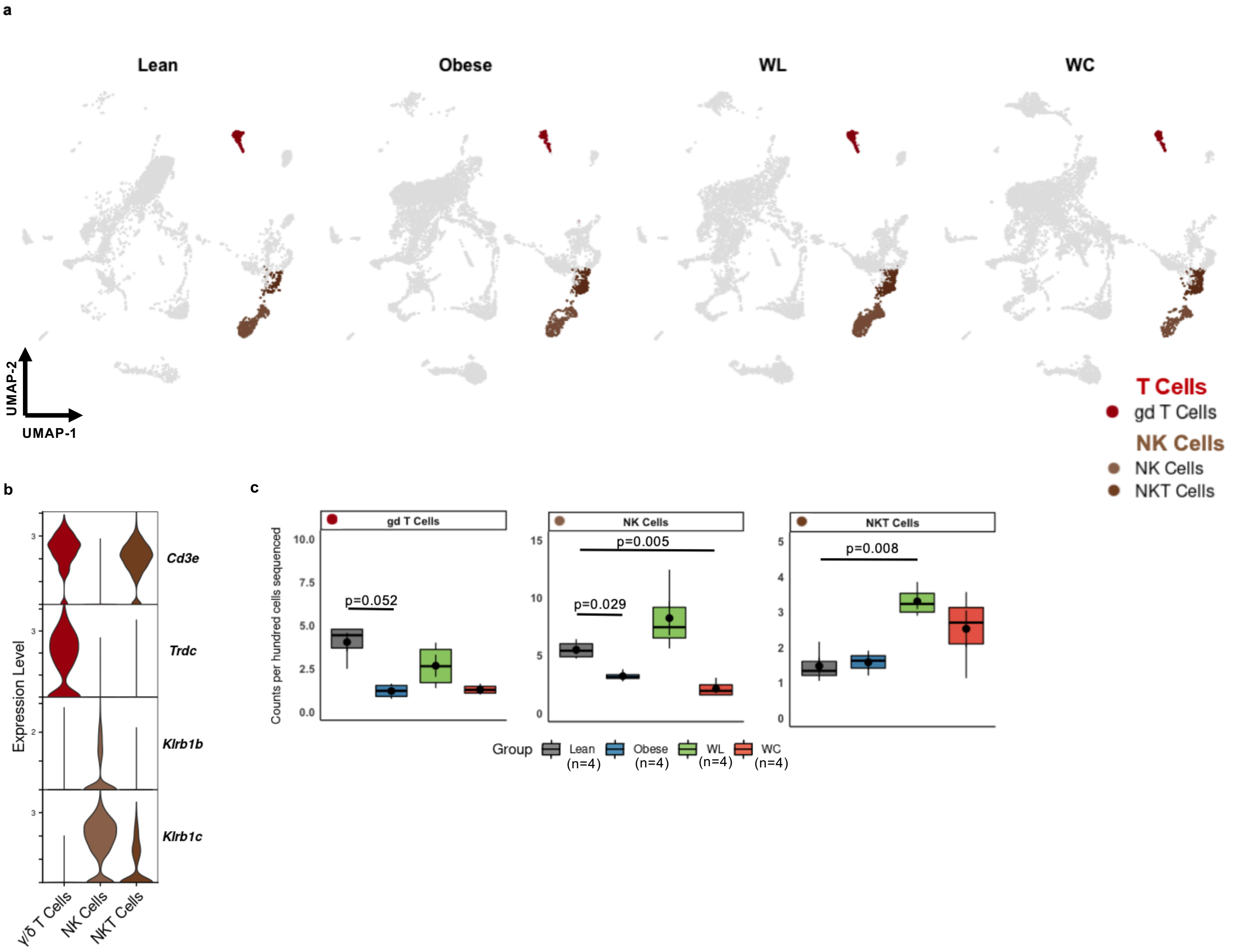

Supplementary Figure 7. Frequency and markers of adipose tissue γ/δ, NK, and NKT cells. (**a**) UMAP of γ/δ T cells and NK cell subclusters by diet group. (**b**) Expression of markers enriched in γ/δ T cells and NK cell subclusters. (**c**) Counts per hundred cells sequenced for γ/δ T cells and NK cell subclusters (mean ± SEM; n=4 mice). Box indicates interquartile range (25th-75th percentile) with 50th percentile indicated by solid line and mean indicated by large circle. Range of whiskers indicates largest and smallest values within 1.5 times the interquartile range and values outside of the range are indicated by small circles. Pairwise two-tailed t-tests with Bonferroni correction for multiple comparisons were used to compare groups for cell counts; significant p values shown.

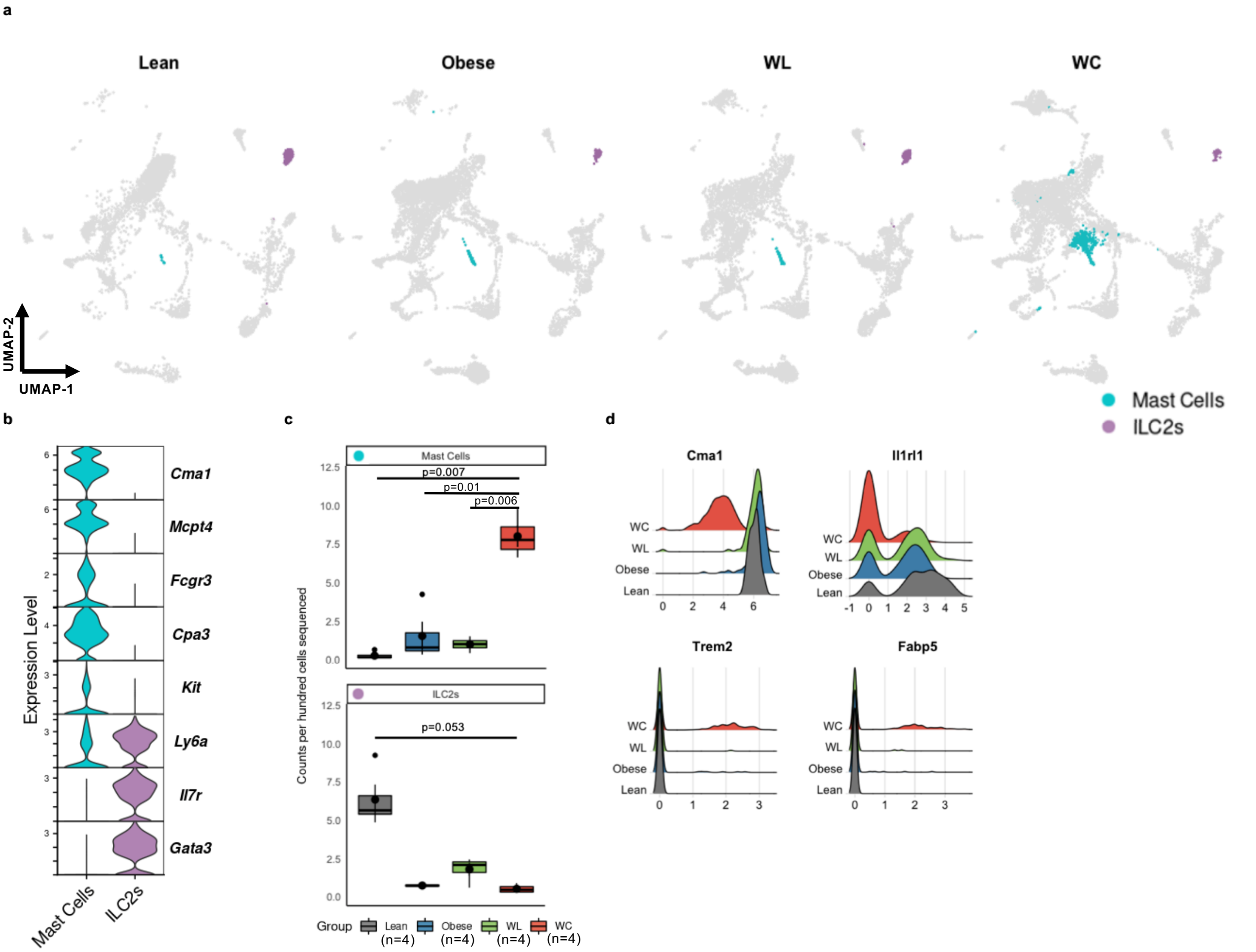

Supplementary Figure 8. **Adipose tissue mast cells have reduced expression of *Ilr1l1* and ILC2s are reduced in frequency after obesity.** (**a**) UMAP of mast cells and ILC2s by diet group. (**b**) Expression of markers enriched in mast cells and ILC2 subclusters. (**c**) Counts per hundred cells sequenced for mast cells and ILC2s (mean ± sem; n=4 mice). Box indicates interquartile range (25th-75th percentile) with 50th percentile indicated by solid line and mean indicated by large circle. Range of whiskers indicates largest and smallest values within 1.5 times the interquartile range and values outside of the range are indicated by small circles. (**d**) Expression of *Cma1* coding for the secreted protease chymase, *Il1rl1* coding for the IL33-R, and lipid-associated proteins *Trem2*, and *Fabp5* in mast cells by diet group. Pairwise two-tailed t-tests with Bonferroni correction for multiple comparisons were used to compare groups for cell counts; significant p values shown.

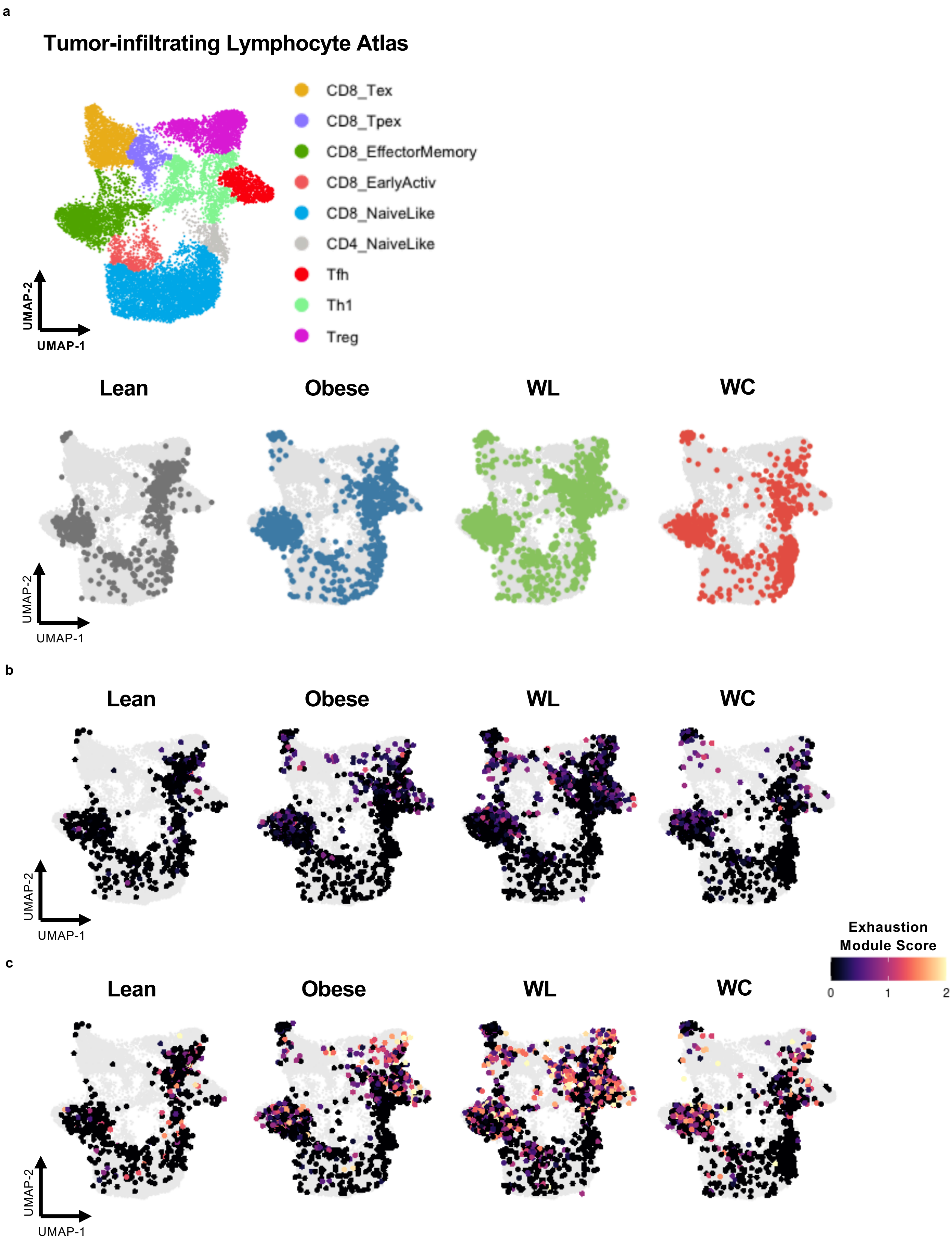

Supplementary Figure 9. Alignment of CD8+ T cells to a tumor-infiltrating lymphocyte reference atlas. (**a**) T cells separated by diet group plotted onto a tumor-infiltrating lymphocyte reference atlas using ProjecTILs. T cells, projected onto the TIL reference atlas, colored by an exhaustion module containing the (**b**) mRNA features *Pdcd1, Tox, Tigit, Lag3, and Entpd1* and **(c)** protein features PD-1 (CD279) and TIGIT. Note that although the reference atlas suggests the majority of cells are effector memory, they express high levels of genes within the T cell mRNA and protein exhaustion modules.

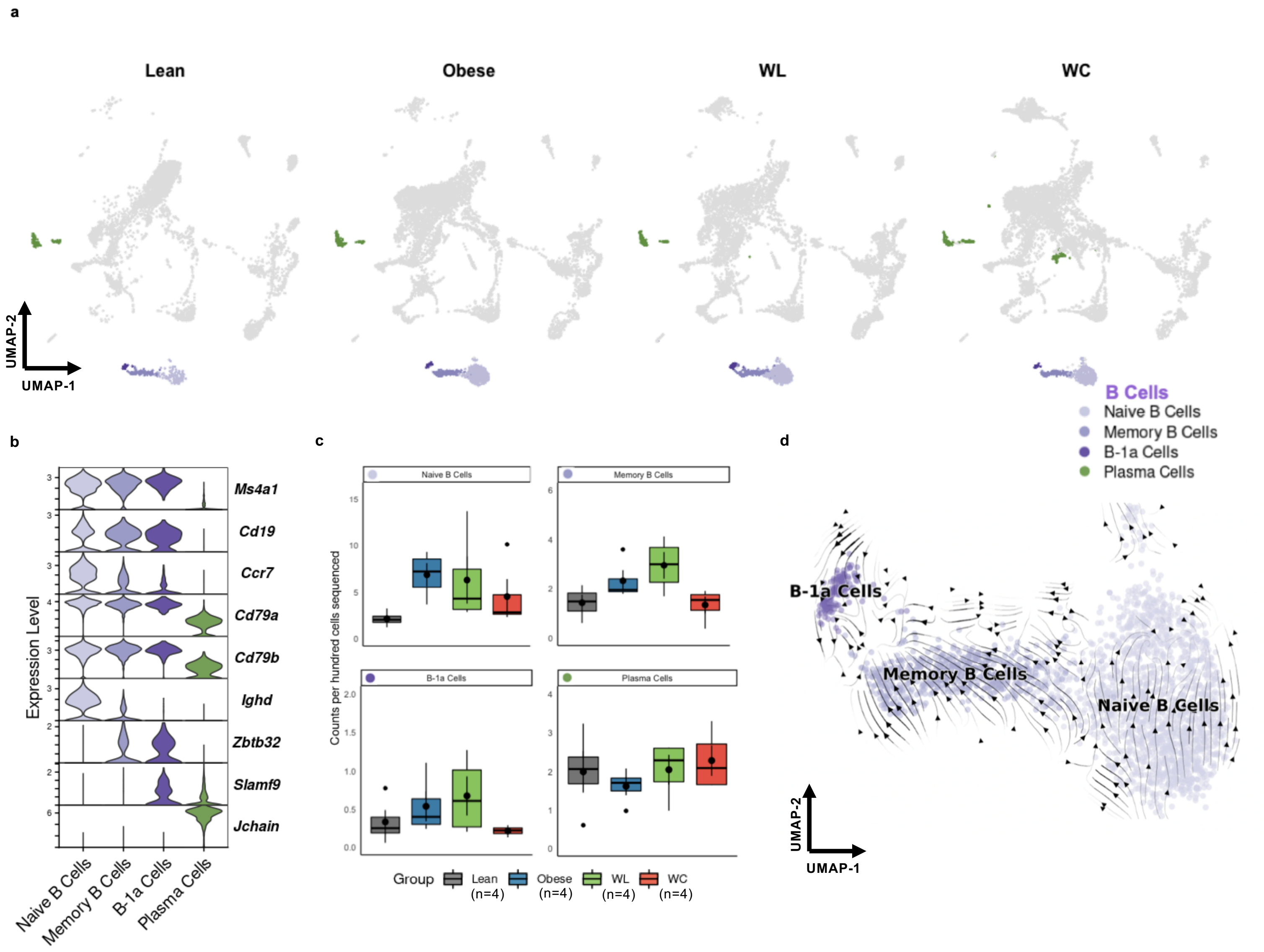

Supplementary Figure 10. Frequency and markers of adipose tissue B cells and plasma cells. (**a**) UMAP of B cell subclusters and plasma cells by diet group. (**b**) Expression of markers enriched in B cell subclusters and plasma cells. (**c**) Counts per hundred cells sequenced for B cell subclusters and plasma cells (mean ± SEM; n=4 mice). Box indicates interquartile range (25th-75th percentile) with 50th percentile indicated by solid line and mean indicated by large circle. Range of whiskers indicates largest and smallest values within 1.5 times the interquartile range and values outside of the range are indicated by small circles. **(d)** Embedding of RNA velocity displayed on the UMAP for B cell subsets. Pairwise two-tailed t-tests with Bonferroni correction for multiple comparisons were used to compare groups for cell counts (no significant differences observed for *adj* $p < 0.05$).

Mouse Info	Hashing Antibody	Pooled Sample
Mouse 1, Lean	0301 anti-mouse Hashtag 1	1
Mouse 2, Lean	0302 anti-mouse Hashtag 2	1
Mouse 3, Lean	0303 anti-mouse Hashtag 3	1
Mouse 4, Lean	0304 anti-mouse Hashtag 4	1
Mouse 5, Obese	0305 anti-mouse Hashtag 5	2
Mouse 6, Obese	0306 anti-mouse Hashtag 6	2
Mouse 7, Obese	0308 anti-mouse Hashtag 8	2
Mouse 8, Obese	0309 anti-mouse Hashtag 9	2
Mouse 9, WL	0301 anti-mouse Hashtag 1	3
Mouse 10, WL	0302 anti-mouse Hashtag 2	3
Mouse 11, WL	0303 anti-mouse Hashtag 3	3
Mouse 12, WL	0304 anti-mouse Hashtag 4	3
Mouse 13, WC	0305 anti-mouse Hashtag 5	4
Mouse 14, WC	0306 anti-mouse Hashtag 6	4
Mouse 15, WC	0308 anti-mouse Hashtag 8	4
Mouse 16, WC	0309 anti-mouse Hashtag 9	4

Cell Sorting Reagents	Manufacturer	Catalog
Fc Block	BD Biosciences	553142
CD45 microbeads	Mitenyi	130-052-301
DAPI	Thermofischer	62247

Hashing Antibodies	Manufacturer	Catalog
0301 anti-mouse Hashtag 1	Biolegend	155861
0302 anti-mouse Hashtag 2	Biolegend	155863
0303 anti-mouse Hashtag 3	Biolegend	155865
0304 anti-mouse Hashtag 4	Biolegend	155867
0305 anti-mouse Hashtag 5	Biolegend	155869
0306 anti-mouse Hashtag 6	Biolegend	155871
0308 anti-mouse Hashtag 8	Biolegend	155875
0309 anti-mouse Hashtag 9	Biolegend	155877

Total Seq C Feature Antibodies	Manufacturer	Catalog
anti-mouse Mac2/gal3	Biolegend	125423
anti-mouse CD279.PD-1	Biolegend	109127
anti-mouse FCyR1	Biolegend	139327
anti-mouse CD4	Biolegend	100571
anti-mouse CCR7/CD197	Biolegend	120131
anti-mouse CD80	Biolegend	109127
anti-mouse CD11c	Biolegend	117361
anti-mouse CD44	Biolegend	103063
anti-mouse NK1.1	Biolegend	108765
anti-mouse TCRy/d	Biolegend	118141
anti-mouse CD39	Biolegend	143815
anti-mouse CD19	Biolegend	115571
anti-mouse CD11b	Biolegend	101275
anti-mouse CD3	Biolegend	100263
anti-mouse TIGIT/vstm3	Biolegend	142119
anti-mouse CD8a	Biolegend	100785

Supplementary Table 1: Ordering Information for antibodies and reagents
