# Supplementary material for: Multiomics reveals persistence of obesity-associated immune cell phenotypes in adipose tissue during weight loss and weight regain in mice
Source: Nat Commun. 2022 May 26;13:2950. doi: 10.1038/s41467-022-30646-4 (PMC9135744; doi:10.1038/s41467-022-30646-4)
Supplement: Supplementary file 3 — Reporting Summary [file 41467_2022_30646_MOESM3_ESM.pdf]

nature portfolio

Corresponding author(s): Alyssa H. Hasty

Last updated by author(s): Aug 12, 2021

Reporting Summary

Nature Portfolio wishes to improve the reproducibility of the work that we publish. This form provides structure for consistency and transparency in reporting. For further information on Nature Portfolio policies, see our Editorial Policies and the Editorial Policy Checklist.

Statistics

For all statistical analyses, confirm that the following items are present in the figure legend, table legend, main text, or Methods section.

n/a	Confirmed	
☐	☒	The exact sample size (*n*) for each experimental group/condition, given as a discrete number and unit of measurement
☐	☒	A statement on whether measurements were taken from distinct samples or whether the same sample was measured repeatedly
☐	☒	The statistical test(s) used AND whether they are one- or two-sided<br>*Only common tests should be described solely by name; describe more complex techniques in the Methods section.*
☒	☐	A description of all covariates tested
☐	☒	A description of any assumptions or corrections, such as tests of normality and adjustment for multiple comparisons
☐	☒	A full description of the statistical parameters including central tendency (e.g. means) or other basic estimates (e.g. regression coefficient) AND variation (e.g. standard deviation) or associated estimates of uncertainty (e.g. confidence intervals)
☐	☒	For null hypothesis testing, the test statistic (e.g. *F*, *t*, *r*) with confidence intervals, effect sizes, degrees of freedom and *P* value noted<br>*Give P values as exact values whenever suitable.*
☒	☐	For Bayesian analysis, information on the choice of priors and Markov chain Monte Carlo settings
☒	☐	For hierarchical and complex designs, identification of the appropriate level for tests and full reporting of outcomes
☒	☐	Estimates of effect sizes (e.g. Cohen's *d*, Pearson's *r*), indicating how they were calculated

Our web collection on statistics for biologists contains articles on many of the points above.

Software and code

Policy information about availability of computer code

Data collection	Leica LASX (v3.7.4.23463) was used to capture immunofluorescence images. ImageJ (v2.1.0/1.53c) was used to quantify lipid droplet size.
Data analysis	All data analysis was performed in R version 4.1.0. FastQ files were processed using CellRanger V3. CellRanger outputs were processed using Velocyto V0.17 and SoupX V1.5.2. Our data was preprocessed using Seurat v4. RNA velocity was estimated and plotted using scVelo V0.2.3 using Reticulate V1.22. DoubletFinder V3 was used to detect heterotypic doublets and further confirm singlets. Cluster annotation was completed with SingleR V1.6.1 with the Immgen and MouseRNAseq databases from the celldex package V1.2.0. For pseudobulk analysis on our web-based tool, the likelihood ratio test in DESeq2 V1.32.0 was used. The miloR 1.0.0 and scProportionTest V0.0.9000 packages were used to display differential abundance testing results. ProjecTILs V1.0.0 was used to map cells to the reference LCMV and TIL atlas. Macrophage polarization and differentiation indexes were plotted using MacSpectrum V1.0.1 in R. We provide open access to example code used to generate our processed object (including pre-processing, data integration, and cell type annotation), a fully integrated Seurat v4 data object, and code used to reproduce all of the figures included in the manuscript via the NCBI GEO (accession #: GSE182233) and two GitHub links: 1)https://github.com/HastyLab/Multiomics-WeightCycling-Vignettes and 2) https://github.com/HastyLab/Multiomics-WeightCycling-Figures.

For manuscripts utilizing custom algorithms or software that are central to the research but not yet described in published literature, software must be made available to editors and reviewers. We strongly encourage code deposition in a community repository (e.g. GitHub). See the Nature Portfolio guidelines for submitting code & software for further information.

Data

Policy information about availability of data

All manuscripts must include a data availability statement. This statement should provide the following information, where applicable:

- Accession codes, unique identifiers, or web links for publicly available datasets
- A description of any restrictions on data availability
- For clinical datasets or third party data, please ensure that the statement adheres to our policy

Raw sequencing files, processed data matrices, UMAP and PCA embeddings, cell metadata, and a fully integrated Seurat v4 R data object are available via the NCBI GEO with the primary accession code (GSE182233).

Field-specific reporting

Please select the one below that is the best fit for your research. If you are not sure, read the appropriate sections before making your selection.

☒ Life sciences ☐ Behavioural & social sciences ☐ Ecological, evolutionary & environmental sciences

For a reference copy of the document with all sections, see nature.com/documents/nr-reporting-summary-flat.pdf

Life sciences study design

All studies must disclose on these points even when the disclosure is negative.

Sample size	12 mice per group was determined to be sufficient to detect difference in ipGTT based on power analysis of our previously published results. We used the ipGTT area under the curve (50,000 +/- 9000 in obese vs. 62,000 in WC) with alpha= 0.05 and 90% power. Four mice per diet group were randomly selected and pooled prior to sequencing, which we felt was a good balance between biological replicates and ultimate cell number (~5,000 cells per mouse).
Data exclusions	As planned during experimental design, 8 mice were excluded from data analysis in Figure 1 because they were used for pancreas phenotyping that will go in another paper using our weight cycling model. Additionally, cells were excluded from single cell analysis if they had less than 200 gene features, less than 500 RNA sequences, or more than 5% mitochondrial RNA content as pre-established based on standard cut-offs found in the literature.
Replication	Figure 1 shows that our metabolic phenotype is reproducible across 6 cohorts of mice (over 4 years and three different scientists). Biological replicates submitted for single cell sequencing show that our group differences are not driven by individual mice. All of the code necessary to reproduce our data set and figures are available on Github: 1)https://github.com/HastyLab/Multiomics-WeightCycling-Vignettes and 2) https://github.com/HastyLab/Multiomics-WeightCycling-Figures.
Randomization	All cages of mice were randomly assigned to cages upon arrival from Jackson Labs by our Department of Animal Care staff. Cages were housed in the same room, but randomly assigned to diet groups prior to the start of diet feeding.
Blinding	Blinding for animal care was not possible due to weekly feeding/weighing of food and body mass. In addition, blinding was not carried out during sample isolation and processing because pooling of biological replicates was necessary. Our core facility was blind to sample groups during processing and subsequent sequencing. Data analysis was carried out on a single integrated data set so all biological replicates were treated equally for downstream characterization.

Reporting for specific materials, systems and methods

We require information from authors about some types of materials, experimental systems and methods used in many studies. Here, indicate whether each material, system or method listed is relevant to your study. If you are not sure if a list item applies to your research, read the appropriate section before selecting a response.

Materials & experimental systems

n/a	Involved in the study	
☐	☒	Antibodies
☒	☐	Eukaryotic cell lines
☒	☐	Palaeontology and archaeology
☐	☒	Animals and other organisms
☒	☐	Human research participants
☒	☐	Clinical data
☒	☐	Dual use research of concern

Methods

n/a	Involved in the study	
☒	☐	ChIP-seq
☒	☐	Flow cytometry
☒	☐	MRI-based neuroimaging

Antibodies

Antibodies used	Fc Block BD Biosciences (Cat#: 553142)<br>CD45 microbeads Mitenyi (Cat#: 130-052-301)<br>0301 anti-mouse Hashtag 1 Biolegend (Cat#: 155861)<br>0302 anti-mouse Hashtag 2 Biolegend (Cat#: 155863)<br>0303 anti-mouse Hashtag 3 Biolegend (Cat#: 155865)<br>0304 anti-mouse Hashtag 4 Biolegend (Cat#: 155867)<br>0305 anti-mouse Hashtag 5 Biolegend (Cat#: 155869)<br>0306 anti-mouse Hashtag 6 Biolegend (Cat#: 155871)<br>0308 anti-mouse Hashtag 8 Biolegend (Cat#: 155875)<br>0309 anti-mouse Hashtag 9 Biolegend (Cat#: 155877)<br>anti-mouse MAC2/GAL3 Biolegend (Cat#: 125423)<br>anti-mouse CD279/PD-1 Biolegend (Cat#: 109127)<br>anti-mouse FCyR1 Biolegend (Cat#: 139327)<br>anti-mouse CD4 Biolegend (Cat#: 100571)<br>anti-mouse CCR7/CD197 Biolegend (Cat#: 120131)<br>anti-mouse CD80 Biolegend (Cat#: 109127)<br>anti-mouse CD11c Biolegend (Cat#: 117361)<br>anti-mouse CD44 Biolegend (Cat#: 103063)<br>anti-mouse NK1.1 Biolegend (Cat#: 108765)<br>anti-mouse TCRy/d Biolegend (Cat#: 118141)<br>anti-mouse CD39 Biolegend (Cat#: 143815)<br>anti-mouse CD19 Biolegend (Cat#: 115571)<br>anti-mouse CD11b Biolegend (Cat#: 101275)<br>anti-mouse CD3 Biolegend (Cat#: 100263)<br>anti-mouse TIGIT/VSTM3 Biolegend 142119)<br>anti-mouse CD8a Biolegend (Cat#: (Cat#: 100785)<br>Rabbit anti-Perilipin-1 (Abcam #9349T; Clone D1D8)<br>Goat anti-Rabbit IgG conjugated to AF647 (Abcam #ab150079)
Validation	All antibodies are commercially available and thus validation was performed per manufacturers workflows. Per Biolegend: Antibody clones are tested for applicable assays in development and then on 1-3 cell types with a positive and negative control, for similar MFI intensity, and validated by QC testing with serial titrations (https://www.biolegend.com/en-us/quality/quality-control). Per BD: "All flow cytometry reagents are titrated on positive and negative cell types... each reagent is bottled to match the previous lot MFI" (https://www.bdbiosciences.com/en-us/products/reagents/flow-cytometry-reagents/research-reagents/quality-and-reproducibility). Per Miltenyi: Antibodies are validated for reproducibility, specificity, and sensitivity (https://www.miltenyibiotec.com/US-en/products/macs-antibodies/antibody-validation.html). Additionally, for CITE-sequencing we titrated identical clones using a PE-tagged antibody for optimal dilutions.

Animals and other organisms

Policy information about studies involving animals; ARRIVE guidelines recommended for reporting animal research

Laboratory animals	Male C57Bl/6J mice from The Jackson Laboratory purchased at 7 weeks of age and acclimated to our mouse facility for one week. Diet was started when mice were 8 weeks old (1 week after arrival). All animals were housed four mice per cage with ad libitum access to food and water at ~23 degrees celcius, ~30% humidity, and with the standard 12hr light-dark cycle.
Wild animals	No wild animals were used in these studies.
Field-collected samples	No field samples were used in these studies.
Ethics oversight	All experiments were approved by the Vanderbilt IACUC committee, protocol number V1800143-00.

Note that full information on the approval of the study protocol must also be provided in the manuscript.
